# Popliteal artery transection associated with a minimally displaced tibial plateau fracture: a case report and review of the literature

**DOI:** 10.1186/s12891-020-3089-8

**Published:** 2020-01-30

**Authors:** Yan-Wei Liu, Yan-Hui Li, Tiecheng Yu, Tianye Yang, Yuying Li, Lei Tan

**Affiliations:** 1 Department of Orthopedic Trauma, The First Hospital of Jilin University, Changchun, No. 71 Xinmin Street, Changchun, Jilin China; 2 Department of Cardiology and Echocardiography, the First Hospital of Jilin University, Changchun, 130021 China; 3 Department of Plastic and Cosmetic Surgery, the First Hospital of Jilin University, Changchun, 130021 China; 4 Department of Hematology, the First Hospital of Jilin University, Changchun, 130021 China

**Keywords:** Popliteal artery transection, Minimally displaced, Tibial plateau fracture

## Abstract

**Background:**

Poplital artery transection injury is potentially catastrophic, or even life-threatening. Severe traumas, including open fracture, gunshot, stabs, and knee dislocation and complex fracture of proximal tibia or distal femur, are the common causes of high rate of amputation due to popliteal artery trauma. No report mentions vascular injury associated with minimally displaced tibial plateau fracture in adult.

**Case presentation:**

A 30-year-old male presented with popliteal artery transection injury associated with minimally displaced tibial plateau fracture. He presented to emergency department, 6 h after fall from ground into a 1-m height hole. Physical examination suggested acute ischemia, with signs of paleness, coldness, anesthesia, hemorrhagic bullae below the right knee level. There was severe swelling and ecchymosis in popliteal fossa and around the leg with significant calf tenderness and pedal edema. Tibialis posterior, dorsalis pedis, and popliteal arterial pulses were not palpable. Radiograph suggested minimally displaced tibial plateau fracture with no evidence of knee dislocation. The patient was taken up for emergency surgery after consultation with vascular surgeon. During the closed reduction external fixation and compartment decompression, popliteal artery trunk was found transected and end-to-end repair was performed. During the post-operational period, no complication was developed and the patient was followed-up for 1 year. At the one-year follow-up, he acquired good stability of his right knee with full range of motion.

**Conclusion:**

Significant swelling and ecchymosis should alert the surgeons to the possibility of vascular injury in knee joint injury, even if there is no fracture or dislocation, or fracture is minimally displaced.

## Background

Poplital artery transection injury can have devastating consequences in patients, as irreversible ischemia can occur in as short as 6 to 8 h. The most common cause of popliteal artery injuries is associated with open wounds-such as those from gunshot, stabs, open fractures or operations [1]. The second most common cause is the closed injuries associated with displaced fractures or dislocations, though some authors report injuries produced by blunt trauma [2]. Although popliteal artery transection complicating a non-displaced proximal tibial epiphysis fracture has been reported [3], no report mentions vascular injury associated with tibial plateau fracture in the absence of displacement or dislocation in adult.

In this case report, we presented a single case of 30-year-old male with popliteal artery transection injury associated with minimally displaced tibial plateau fracture, was treated in time and saved the affected limb successfully.

## Case presentation

A 30-year-old male (body mass index, 28.8 kg/m2) presented to our emergency department, 6 h after fall from ground into a 1-m height hole, with an isolated struck injury of the right flexed knee while he was drunk. According to his parents’ description, he walked for a short distance. He complained acute pain in his right knee when admitted.

In the immediate physical examination of the patient, the right knee level showed clear evidence of acute ischemia, with signs of paleness, coldness, anesthesia, hemorrhagic bullae. There was severe swelling and ecchymosis in popliteal fossa and around the leg, and there was significant calf tenderness and pedal edema. Tibialis posterior, dorsalis pedis, and popliteal arterial pulses were not felt, and capillary refill and pinprick bleeding were delayed. There was severe pain with passive stretch of the muscles when the toes or foot are plantar flexed.

**Fig. 1:**
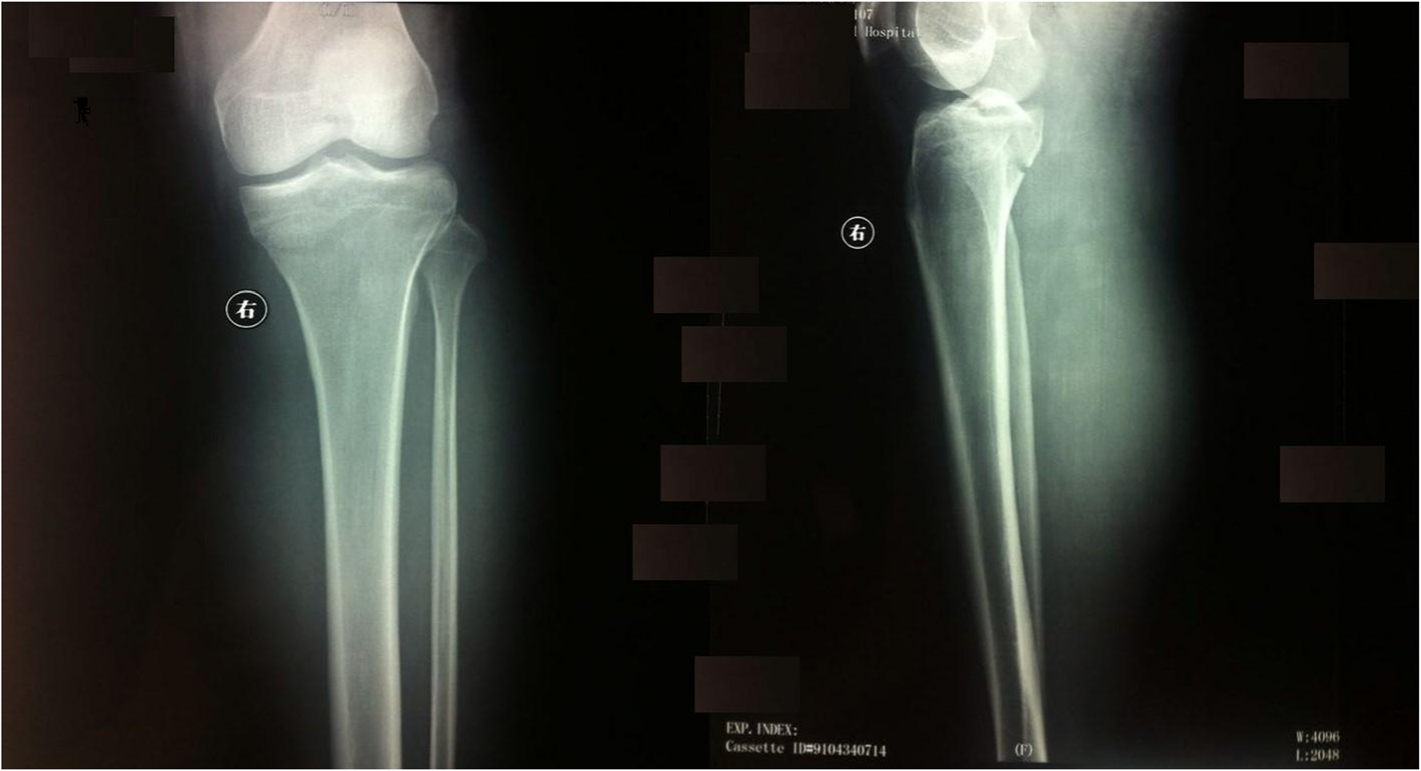
An oblique fracture, involving the tibial spines, medial and lateral surfaces of the tibal plateau illustrated in an anteroposterior (left), and lateral radiographs (right)

Radiograph was taken which showed minimally displaced tibial plateau two columns fracture with no evidence of knee dislocation, but significant soft tissue swelling (Fig. 1). After consultation with vascular surgeon, we believed that there was a high probability of popliteal artery injury. The patient was sent to operation immediately.

**Fig. 2:**
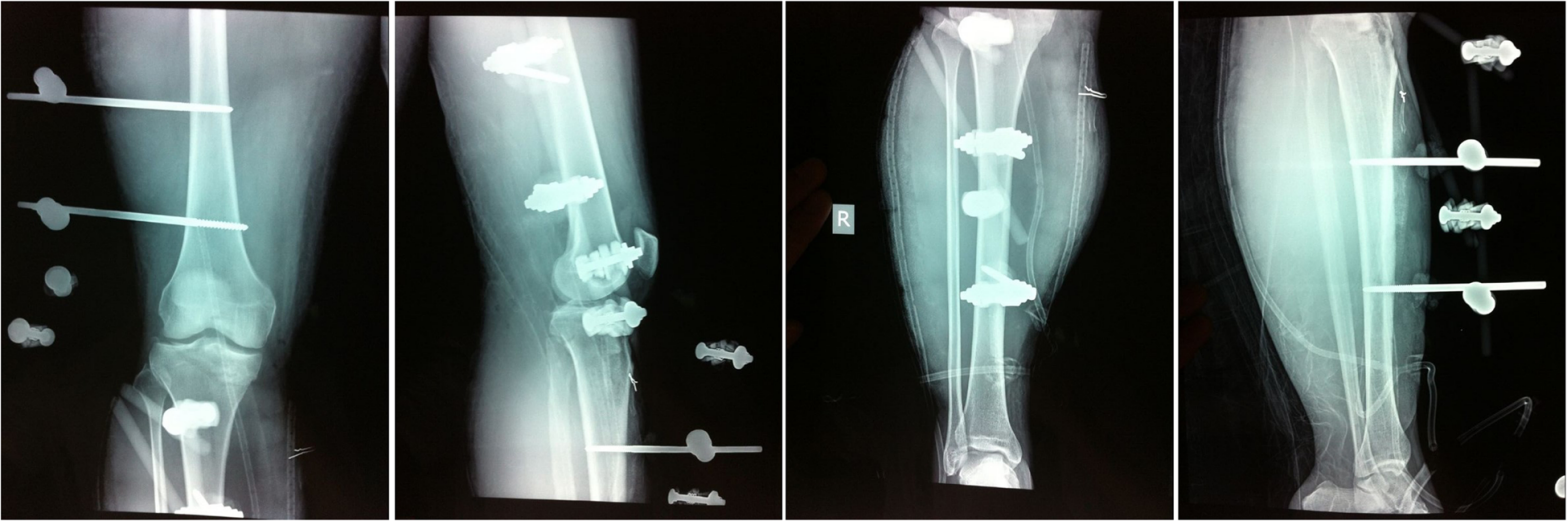
Postoperative radiographs. The fracture was fixed with external fixator

**Fig. 3:**
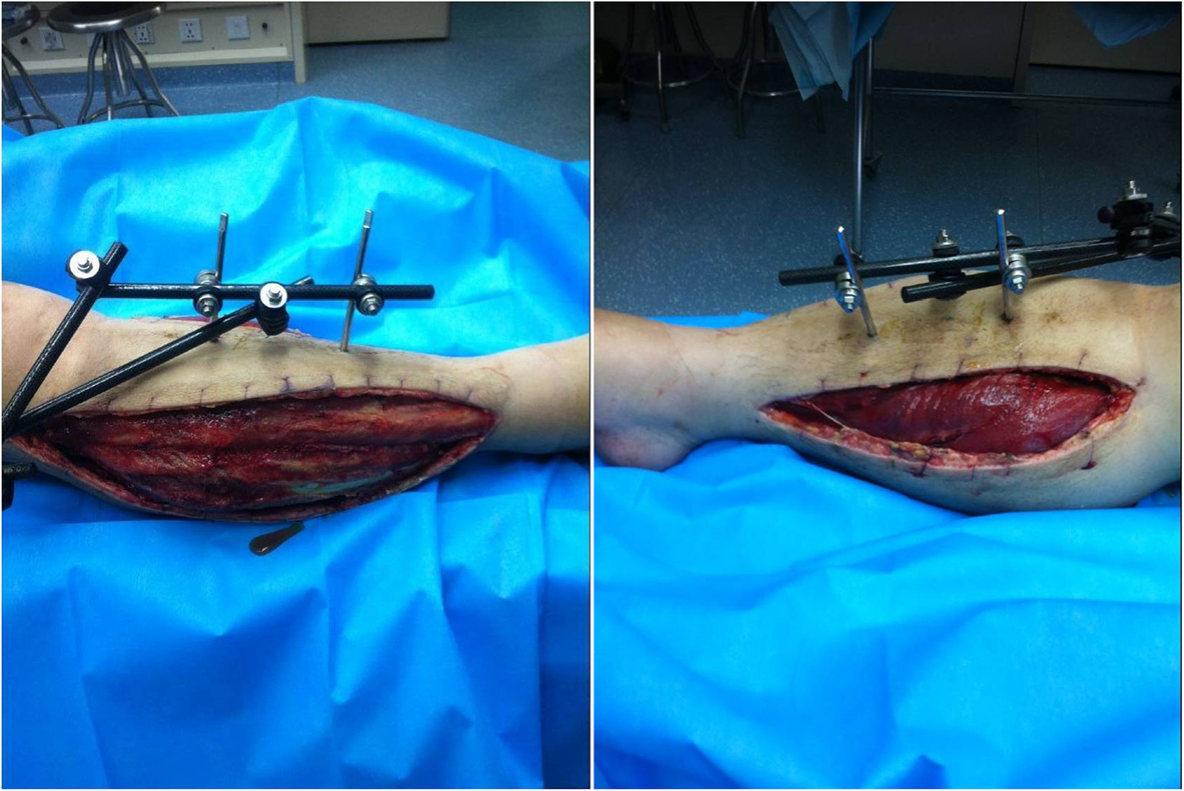
Intraoperative picture of lateral(left) and medial(right) incision of compartment decompression

**Fig. 4:**
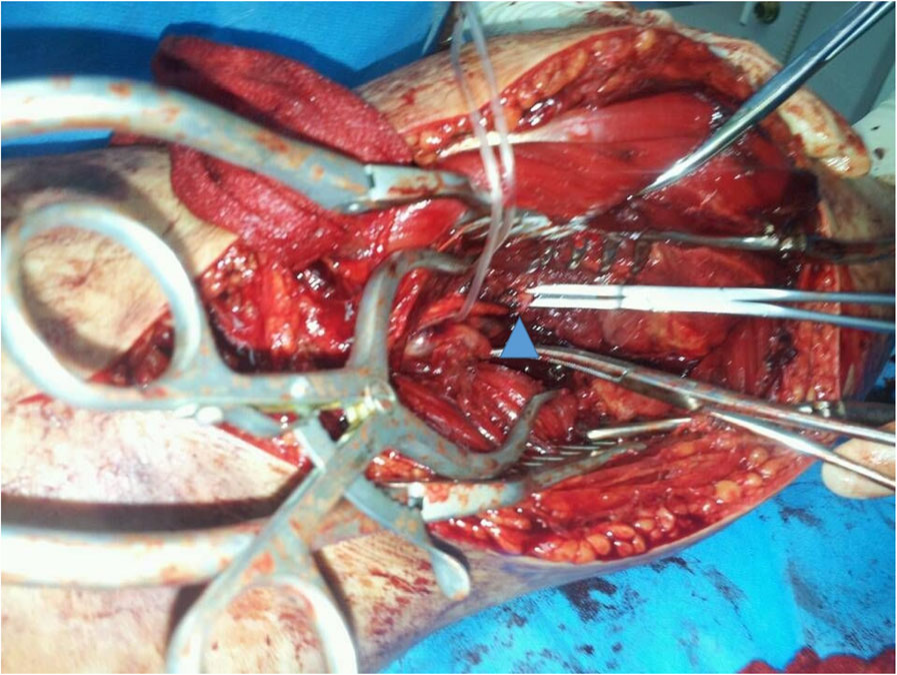
Popliteal artery transection (blue triangle) was observed intraoperatively

Emergency surgery was performed to salvage the ischemic limb at 6.5 h after injury. The patient was placed in the supine position under general anesthesia. Knee ligaments examinations did not reveal any pathological finding. Closed reduction was performed and fixed using 2 femoral lateral-medial Scanz pins and 2 tibial anterior-to-posterior Scanz pins, and the external fixator was connected with the use of rods and clamps (Fig. 2). Because the patient developed compartment syndrome, compartment release was performed through double-incision fasciotomy and the wounds were covered by vacuum-assisted closure (Fig. 3). While exploring the popliteal artery of the right knee, popliteal artery trunk was found transected (Fig. 4) and thus end-to-end repair was done by a vascular surgery team. Slightly decreased dorsalis pedis arterial pulses were recovered immediately postoperatively. The patient was transfused 2 units of blood for hematocrit < 26%.

Twelve hours after surgery, the anticoagulant was started. Heparin intravenous infusion was given for 5 days and rivaroxaban for 3 months. Debridement was performed again 2 days after the first operation, and the devitalized peroneus longus was removed. Fasciotomy wound was closed on the 14th day after surgery. The patient refused to be treated with internal fixation and was discharged after 4 weeks of inpatient treatment with satisfactory distal limb perfusion. Follow-up was carried out in the outpatient department. The external fixator was removed and knee mobilization started at 12 weeks. Without strict rehabilitation schedule, the patient recovered very well although foot drop had not been improved. The neurological examination was checked and common peroneal nerve injury was excluded. During the post-operation follow-up, no complication was developed in the wound. At the 1 year follow up, the patient acquired good stability of his right knee with full range of motion. During the whole treatment process, the patient was very satisfied with the treatment and recovery.

## Discussion and conclusion

Popliteal artery transection injury is a condition resulting in limb necrosis or amputation that can be potentially life-threatening [4]. Severe trauma, including open fracture, gunshot wounds, stabs, knee dislocation and complex fracture of the proximal tibia or distal femur are the principal causes of the high rates of amputation in popliteal artery trauma (Table 1) [5–15]. There have been no reports of vascular injury associated with tibial plateau fracture in the absence of displacement or dislocation in adults.

**Table 1:** The causes of popliteal artery injury

Authors	Open injury			Close injury	
	Fracture/dislocation	Gunshot	Stab	knee dislocation	Displaced fracture
Leclerc et al. 2018 [5]	20	3			
Wagner et al. 1994 [6]		99	7		
Nicandri et al. 2010 [7]				6	
Miranda et al. 2002 [8]				6	
Papadopoulos et al. 2006 [9]					1
Hesse et al. 2006 [10]					1
Klineberg et al. 2004 [11]				12	
Shinomiya et al. 2018 [12]					1
Bonnevialle et al. 2006 [13]	5			5	1
Steele et al. 2012 [14]				1	
Treiman et al. 1992 [15]				27	

The distal portion of the popliteal artery lies close to the posterior aspect of the proximal tibia, where the firm connective tissue septa maintains the position of the vessel against the knee capsule. Therefore, the popliteal artery is fixed proximally to the popliteal fossa. Once displaced fractures or dislocations occur, the popliteal artery has no buffering room and so becomes vulnerable to injury. The mechanisms of popliteal artery transection injury caused by minimally displaced proximal tibial fracture may be due to the presence of transient fracture dislocation, the patient’s autonomic response after injury possibly leading to spontaneous reduction of the fracture.

Rapid diagnosis and surgery decrease the period of ischemia and thus, the rate of amputation. A common means of diagnosis of popliteal artery injury is Doppler ultrasound, the gold standard being angiography [11]. However, both methods are time consuming. Time is the most important factor that determines whether a patient’s limb can be saved, the literature indicating that limb ischemia of a duration greater than 6 h results in the proportion of necrosis or amputation in limbs likely to reach 30% [16]. For each additional hour, this rate increases by 12% [17]. Subasi et al. suggested that physical examination findings, such as bleeding from a penetrating wound, pulsatile hematoma, or the absence of a distal pulse, are sufficient to establish a diagnosis of the injury [18]. Therefore, following consultation with a vascular surgeon in cases such as these, patient are transferred directly to the operation theater 30 mins after arrival in the emergency department. This greatly saves time, contributing to eventual preservation of the affected limb.

Even if a pulse was detected, popliteal artery injury should be considered, as there may be perfusion anomalies. In such cases, measurement of the ankle-brachial index (ABI) is the subsequent consideration in suspected cases. Patients with an ABI ≥0.9 may be observed. For an ABI below 0.9, CT, angio or Doppler are required, followed by immediate vascular exploration and repair if positive findings so mandate [19]. We did not utilize these methods as the case was clinically apparent by the absence of a pulse.

There are a number of commonly recognized fracture patterns that involve either the medial or lateral tibial condyle. Tibial plateau fractures that involve the medial plateau, as in the present case, exhibit an increased risk of development of compartment syndrome and neurovascular injury compared with the lateral counterpart [20].

Any delay in treatment of acute compartment syndrome can be catastrophic, leading to serious complications such as permanent sensory and motor deficit, contractures, infection, and at times, amputation of the limb [21–23]. If there is clinical presentation such as pain with passive stretch of the muscles, paresthesia, paralysis and lack of a pulse, the fascia compartment should be decompressed thoroughly as soon as possible. If the clinical presentation is not so apparent, and duration of limb ischemia is greater than 5 h, preventive fasciotomy should be performed to avoid reperfusion injury.

Given below are a few recommendations regarding this case. Firstly, surgeons should pay attention to the possibility of vascular damage in knee joint injury, even if there is no fracture or dislocation, or the fracture is minimally displaced. Secondly, auxiliary examination should not be relied upon, and the observation that saved time saves limbs should be remembered. Thirdly, for suspicious injuries whose symptoms are not apparent, clinical examination using the ABI is valuable as an alternative. Fourthly, fascia compartment decompression should be performed using quick thinking and decisive action.

In conclusion, we have reported a rare case of popliteal artery transection injury with minimally displaced tibial plateau fracture. As popliteal artery transection injury is potentially catastrophic, the possibility of vascular injury should be considered in knee joint injury, even if there is no fracture or dislocation, or when the fracture is minimally displaced. If this occurs, the patient should be treated immediately and thoroughly.

## Data Availability

The dataset supporting the conclusions of this article is included within the article.

## Abbreviation

ABI: Ankle-brachial index
